# Enhancing antenatal education in Pakistan: an audit and recommendations

**DOI:** 10.1186/s12905-023-02799-x

**Published:** 2023-12-04

**Authors:** Maliha Abbas, Shelina Bhamani, Yasmin Kanjani, Lumaan Sheikh

**Affiliations:** Department of Obstetrics and Gynecology, Aga Khan University, Karachi, 74800 Pakistan

**Keywords:** Antenatal, Education, Parents, Curriculum

## Abstract

**Background:**

Antenatal Education equips parents with knowledge for safe maternal health and infant care. It also reduces fear and anxiety during childbirth. ANE curriculum can vary according to country and institute. It can include classes focusing on childbirth, pain relief techniques, mode of birth, parenting, breastfeeding, breathing techniques, etc. Although ANE is widely practiced in developed countries, there is no standard program in developing countries like Pakistan. This study aims to improve antenatal education at a tertiary care hospital in Karachi, Pakistan potentially proposing an upgraded curriculum as a national standard.

**Methods:**

This multiphase study used mix-method design was conducted in the Obstetrics and Gynaecology Department of a tertiary care hospital of Karachi, Pakistan from 2019 to 2021. Phase 1 of the study included reviewing and comparing the hospital’s antenatal curriculum with existing literature, followed by Phase 2, which was a desk review of attendance and patient feedback. The 3^rd^ phase involved IDIs (in depth interviews) from health care workers (Obstetrics experts) to understand their perspectives regarding the ANE and the conducted classes. For phase one, gaps were identified and reported theoretically. For phase two, the annual attendance was recorded and participants’ satisfaction with the classes assessed. Qualitative data from phase 2 and 3 was converted into themes and sub-themes.

**Results:**

The audit showed a decline in the attendance of antenatal classes due to the pandemic and consequent shift to online sessions. The low attendance in online courses could be attributed to various factors. Patient feedback was generally positive, with a majority expressing high satisfaction levels. Expert feedback highlighted the need for additional topics such as mental health and COVID in pregnancy, as well as fathers' involvement. The curriculum was updated to include these topics and made more interactive with printed handouts for parents.

**Conclusion:**

A standardized antenatal education covering various topics surrounding pregnancy, childbirth, and postnatal care must be available to parents nationwide.

## Introduction

Pregnancy is a time of significant physiological and emotional change, and childbirth remains a source of great fear and concern for a woman having a baby [1, 2]. Inadequate knowledge can propagate fear and detrimental habits amongst this vulnerable population. Antenatal Education (ANE) was hence introduced to equip parents with the necessary information to make educated, safe decisions regarding maternal health, antenatal care, labor, postnatal care, and infant care, as well as allow them to get the necessary parenting advice, share their experiences and get their questions answered [3–5].

The curriculum components for antenatal classes can vary according to country and institute. It can include classes focusing on childbirth, pain relief techniques, mode of birth, parenting, breastfeeding, breathing techniques etc. [6]. Furthermore, the content's delivery mode can also vary from lectures to role play education, leaflets, online classes, etc. These teaching sessions are usually conducted by childbirth educators, antenatal doulas, nurses, physiotherapists, or trained educators [7].

Antenatal education has been extensively researched to have a significant positive impact on maternal health and practices. Apart from the apparent impact of decreased anxiety and fear of childbirth and improved confidence and self-sufficiency of mothers, studies have also established an impact on the mode of delivery with antenatal classes leading to decreased cesarean birth rate [5, 8, 9]. Moreover, an inverse relationship between ANE and the use of epidural analgesia and maternal stress has also been established in some studies [10]. Partner involvement throughout pregnancy and childbirth can increase with targeted antenatal lessons. A study conducted in Malaysia also found decreased postnatal depression amongst the population receiving antenatal classes [11]. Furthermore, ANE can also lead to fewer false labor admissions, thus reducing the hospital’s burden and allowing for better allocation of resources [12].

ANE is considered an essential component of holistic antenatal care, steadily gaining traction globally. It has been an established as a standard practice in the developed world for years and is slowly being adopted by the developing world. The World Health Organization (WHO) has recommended antenatal education as a priority intervention to improve birth readiness and preparedness, especially in developing countries [13, 14]. However, no standard antenatal education program exists in most developing countries, including Pakistan. With such low literacy levels and the cultural practices in Pakistan impeding access for women, it is imperative to establish a standard antenatal education program that can improve parents’ knowledge and capability to make decisions regarding pregnancy and childbirth.

In Pakistan, the availability of structured antenatal education is limited to private-sector hospitals, where often, it is integrated into routine checkups instead of well structured educational sessions. The government of Pakistan has implemented a commendable door-to-door program for maternal education, where lady health workers provide antenatal counseling. However, the program does not follow a comprehensive standardized curriculum.

Dissemination of antenatal education must be dynamic, constantly evolving to meet the needs and expectations of pregnant women. This quality improvement project aims to evaluate the antenatal education being provided in the leading tertiary care hospital of Karachi, Pakistan, for its adequacy and up-to-datedness and upgrade it as required. In doing so, we also aim to lay the groundwork for a comprehensive antenatal curriculum for hospitals nationwide.

## Methods

### Study design

An Antenatal Education curriculum-based mixed-methods design was used. The study consisted of three phases with quantitative and qualitative data collection simultaneously. Phase one featured a review of the curriculum and its comparison to other curriculum; phase two involved a desk evaluation of attendance and patient feedback documents and phase three entailed evaluating physician perspectives on the same.

### Study setting

The study setting was a private tertiary care hospital's Department of Obstetrics and Gynaecology (OB-GYN), a largest private tertiary care hospital in Karachi, Pakistan with 128 dedicated beds for OB-GYN conducting approximately 6000 deliveries each year (hospital records).

### Study participants and eligibility criteria

Parents attending the ANE classes and experts in the relevant field i.e., Registered Nurses (RN) and Obstetricians. Participants included who provided the informed written consent.

#### Data collection method and tool

##### Phase 1: Curriculum review

The first phase of the audit included a desk review of all the presentations and teaching material by a team of investigators that included a qualified post graduate MBBS research fellow in maternal health stream, an antenatal nurse expert, and a full-time faculty ECD lead who is a global certified trainer in First 1000 days parenting education. The curriculum was thoroughly vetted and evaluated by the team members individually based on a self-constructed, thorough criteria involving a Likert scale grading of the the following categories: significance, ease of understanding, practical impact, authenticity, relevance of topic, and interactivity of the lesson. the next step involved consolidation of the individual assessments of the team members. For this review, people from the disciplines of women physio, genetics, lactation consultation and nutrition were consulted as needed regarding potential inaccuracies. This desk review was used to detect potential strengths and weaknesses in our ANE curriculum, and to identify the potential target areas to be addressed by the obstetric experts for improvement in the later phase of the study.

##### Phase 2: Attendance and parent feedback

The attendance records of the parents who took ANE classes were reviewed to determine the level of engagement with the curriculum. These were collected by identifying the parents with associated medical records who attended the classes and asking them for feedback if they consented. 41 graded evaluations and 25 subjective patient feedback comments were reviewed to assess the effectiveness of the curriculum. All attendees were given a feedback form to fill out at the end of the last class. This feedback included grading of level of satisfaction with various components of the antenatal classes including clarity of information, learning material, classroom environment, presentation and tour of the hospital facilities. A positive likert scale was used for grading the level of satisfaction i.e., 1 as strongly dissatisfied and 4 as satisfied.

##### Phase 3: Health care providers perspectives

In-depth interview (IDI) guide was designed, and data was collected from two Obstetricians and two registered nurses (RNs) from the department of OB-GYN. The IDI questions focused on their perceptions of the curriculum, including the relevance of the topics covered, the effectiveness of teaching methodologies, and the usefulness of the assessments, focusing especially on the weaknesses identified in phase 1 of the study. Their input regarding any inaccuracies and recommended changes were noted. The data obtained from this phase was used to identify areas of improvement for the curriculum.

### Data analysis

The data was obtained from each phase of the study and further analyzed. For the first phase, gaps were identified and reported theoretically. Phase two analysis involved the number of participants attending each year reported and percentage for the satisfaction levels, for which, 25 patient feedback evaluations were extracted and arranged according to their subject matter. For the qualitative component, data was transcribed and translated to develop codes and later themes and sub-themes were reported. The data was analyzed using SPSS version 24 and NVivo software.

## Results

### Gaps identified in curriculum during phase 1

The first phase of the study identified several important issues missing from the curriculum being used for the antenatal classes. The curriculum was found to be focused on labour and delivery with inadequate emphasis on lifestyle changes required during the course of the pregnancy as well as postnatal care. Topics like immunizations and follow up visits during pregnancy, postnatal contraception, appropriate storage of breastmilk and tackling postpartum depression were not addressed in the curriculum.

Furthermore, the some of the presentations were found to be insufficiently patient-centred for example, explaining the stages of pregnancy and labour according to what the mother should be expecting rather than the physiological details. Furthermore, there was also a lack of appropriate referrals mentioned in the classes for example the handouts given to the participants lacked necessary information on specialized nutritionist clinics available for further guidance regarding diet and the pamphlet for the breastfeeding session missed mentioning referral to lactation clinic if required.

### Trends in attendance over the years in phase 2

The attendance of the antenatal classes conducted from 2019 to 2022 was compared and showed a drastic decline from 2019 to 2021 (Table 1). This was in response to the COVID-19 pandemic, during which, the classes were shifted to an online format. Due to the teaching resources not being very adaptable to a virtual system, online classes took some time to become functional. The low attendance in online sessions could be attributed to internet connectivity issues, technological illiteracy, lack of awareness with limited physical presence around other parents and lack of motivation at home.

**Table 1:** Number of parents attending antenatal classes from 2019–2021

	2019	2020	2021
Yearly attendance of online classes	143	58	36

### Graded quantitative patient evaluation in phase 2

To assess the participants’ satisfaction with various aspects of the ANE being offered, 25 of the evaluation forms were quantitatively analysed as mentioned in Table 2. Majority (83.3%) of the attendees agreed that the topics were presented with knowledge and clarity. Most of them (85.7%) responded that the classes met all their expectations and that they would recommend the classes. There were high levels of satisfaction with method of presentation (78.6%), classroom environment (81%) and the tour of facilities (81%). In comparison only half of the parents were satisfied with the resource materials provided to them in the classes.

**Table 2:** Percentage of attendees and their varying levels of satisfaction with components of antenatal classes

Components	Level of satisfaction (% of responses)			
	1 (Strongly dissatisfied)	2 (Dissatisfied)	3 (Neutral)	4 (Satisfied)
Method of presentation	1	0	19	78.6
Resource materials	1	4.8	42.9	50
Classroom environment	1	0	16.7	81
Tour of facilities	1	0	16.7	81

### Open ended parent feedback

Some of the parents also gave some additional comments and feedback in the evaluations. Antenatal classes received positive feedback for their beneficial preparation and relaxation for delivery, but participants suggested improvements in timing, curriculum, and postnatal education. They recommended shorter, spaced-out classes with comprehensive recaps, practical sessions on baby care, and the inclusion of resources like booklets and information on pricing for better support during the program (Table 3).

**Table 3:** Extracted participants’ comments categorized according to sub-themes

Sub themes	Comments from attendees	n
Patient satisfaction	The antenatal classes are very beneficial and made me much more prepared and relaxed for the delivery	10
	I found the classes very beneficial. The exercises explained were particularly helpful	
	Very useful especially for first time mothers	
	It was a great effort with all necessary information being given and questions being answered very well	
	Overall, a very effective and useful experience	
	These classes are very informative but are not advertised or promoted during hospital visits. I heard about them from someone otherwise would not have known about them	
	Courses were very informative. More courses should be conducted from time to time	
	Very informative programme	
	I am glad that my questions were thoroughly answered	
	Very satisfied with the classes and am very glad I took them	
Structure of classes and curriculum	Class timing is very long for pregnant mothers to sit comfortably	5
	Classes were a little lengthy timing wise, otherwise very helpful	
	Shorter length of classes a greater number spaced out throughout the 9 months would be better	
	Please keep a full course recap in the final week	
	The classes regarding changes in pregnancy, diet and nutrition should be offered earlier in the pregnancy and those concerning labour and delivery towards the end of pregnancy	
Inclusion of Post natal education	Perhaps sessions like changing diapers and baby’s clothes could be very helpful	4
	More courses should be conducted throughout the course of pregnancy, especially about important things to know about after delivery	
	Labour and birth were covered in the classes but I would have liked to know more about the precautions I have to take after birth	
	I think the delivery sessions were very good. A little information about managing a newborn baby, especially from the perspective of a father would have been great	
Resource availability for personal use	Booklets and other additional aid would have been greatly appreciated	6
	Very thorough class. All my queries were answered. Pamphlets should be provided as well	
	A handout of a summary of each class would be very beneficial if provided	
	The presentations should be shared with the participants for viewing at their convenience	
	Costing/pricing for rooms and delivery/pain relief methods would be of help	

### Perceptions of health care providers regarding the quality of the curriculum

According to the expert feedback, the curriculum was lacking some major components. These included a basic introduction to pregnancy and foetal development, first 1000 days of life and importance of talking to baby in the womb a mental health and well-being during pregnancy. As said by one of the experts* “Mental health is such a stigma in our culture, it should be addressed so women can be reassured that it’s a common problem and know when to approach a psychiatrist.”*

Moreover, the consensus of experts was to include a separate class for covid during pregnancy and childbirth as that has become a cause of significant concern in majority of women since the pandemic. Two of the obstetricians addressed this saying:

“With the pandemic, pregnant women are especially concerned and often ill-informed about what COVID during pregnancy can entail. A specific lesson to address the common fears and misconceptions is a need in current times”.

“Pregnant women are usually very apprehensive of vaccinations during pregnancy, especially the COVID vaccines and need reassurance of their safety”.

Furthermore, the curriculum was assessed to be solely targeted to mothers. Due to cultural and economic conditions, there is a distinct lack of partner involvement in antenatal care in Pakistan. The need for including aspects involving the fathers was expressed by all experts as mentioned:* “there is already a distinct lack of paternal involvement during pregnancy in the culture prevalent in Pakistan. Antenatal lessons to specifically include fathers could slowly bring about a change in that”.*

## Discussion

The attendance of antenatal classes being conducted saw a drastic decline from 2019 to 2021. This was most likely due to the COVID-19 pandemic since the lockdown kept parents from being able to take the classes in person, and the existing curriculum was not adaptable to a virtual platform, with the nurses taking the sessions also not being comfortable with the use of technology to conduct the classes online. According to a study conducted in the United Kingdom (UK) during the pandemic, educators found interacting with parents online more difficult and were second-guessing their lessons [15]. However, literature also states that online antenatal education was well used and received by mothers during the pandemic [16]. In fact, one such study established a statistically significant increase in confidence among mothers after digital antenatal classes compared to in-person classes [17]. Therefore, the gap needs to be bridged by providing suitable training to educators, enabling them to conduct sessions on a virtual platform proficiently. This approach will additionally offer the benefit of delivering customized, pre-recorded lessons to parents to address everyone’s pregnancy concerns and inquiries.

One of the themes that emerged from the patient feedback in our study pertained to the necessity for postnatal care education. This finding is consistent with the antenatal education being provided globally, including in developed countries. In a study conducted in Australia, new-born educators found that postnatal care and parenting advice was sought after by parents more than antenatal [18]. Other studies have found a positive relationship between prenatal education and breastfeeding rates, associated nipple trauma, immunization rates, overall quality of life, and antenatal education [19, 20]. Since postnatal care has been found to be grossly underused in Pakistan, it is imperative to target raising awareness during the antenatal period [21].

The experts highly emphasized inclusion of mental health during pregnancy and postpartum. Established risk factors for postpartum depression include illiteracy, low socioeconomic status, access to healthcare and cultural stigma regarding mental health issues and lack of education are prevalent in the country [22, 23]. Not only can antenatal classes educate parents about postpartum depression and relevant coping mechanisms, but better support and preparedness throughout the pregnancy can decrease the incidence of postpartum depression. A study conducted in Japan found that attendees of antenatal classes scored significantly less on the Edinburgh Postnatal Depression Scale than non-attendees [24]. Given that Pakistan exhibits the highest prevalence of postpartum depression among Asian nations, tackling this issue and promoting awareness through any available means is crucial. Antenatal education presents an excellent opportunity to achieve this goal [25].

As mentioned by the experts, antenatal care and education in Pakistan predominantly concentrates on mothers, with little to no partner involvement. The antenatal classes being provided in our institute also had only one session of couple classes, with the rest being catered especially for mothers. However, studies have consistently shown a positive impact of increased partner involvement in antenatal care [26, 27]. A randomized control trial found significantly improved maternal health behaviour with antenatal education involving the couple compared to that of the pregnant women alone [28]. It is imperative to establish antenatal education that intentionally involves male partners as well, especially in a country like Pakistan where cultural norms lead to husbands often making the major decisions in the household. Conducting the lessons at times separate from office hours, as well as involving male teachers, and involving activities that the couple can do together are some of the ways to increase male partner involvement that can be incorporated into the antenatal education.

### Strengths and limitations

This is the first ever quality improvement initiative by a teaching tertiary care university hospital in Pakistan. There are no established programs in Pakistan which tackle the antenatal education in Pakistan. This study allowed for augmentation of the education initiative being conducted at the tertiary care centre, to include previously ignored areas, and improvement of the content, structure and delivery of lessons. This study builds on the limited antenatal education being provided by the centre by comparing it to the standard in existing literature, allowing for a reliable resource to be formed, which can be used as an aid for a standardized curriculum nationwide since none exists currently. This study also used a robust method to understand the perspectives and opinions of both the parents and health care workers, allowing for maximum efficiency of the ANE, and simultaneously audited the existing curriculum to improve it.

The limitations include small sample size and lack of generalizability since this study was conducted at a single centre private tertiary care hospital. Moreover, this study failed to triangulate and connect both qualitative and quantitative data.

## Conclusion

The antenatal Education curriculum should be standardized across the country that can be implemented through a hybrid approach of both online and in-person classes. This approach ensures flexibility and accessibility for all expecting parents. The curriculum should cover a wide range of relevant topics to provide comprehensive education and support throughout the antenatal period. The important components which must be included are pregnancy, foetal development, first 1000 days of life of a baby, nutrition and exercise in pregnancy, labor and modes of pain relief that are available during labour, importance of breastfeeding and basics of new-born care. These educational sessions should be delivered in way that they are interactive for the participants, should address the cultural norms and values, and should encourage participation from both the parents and not only the mother, as is seen in current practice.

## Data Availability

Available on reasonable request to the corresponding author.

## Abbreviations

ANE: Antenatal Education
UK: United Kingdom
WHO: World Health Organization
